# Three-point traction method for endoscopic submucosal dissection using clip-with-thread and clip-with-silicon bands for large early gastric neoplasms

**DOI:** 10.1055/a-2219-8130

**Published:** 2024-01-08

**Authors:** Ryohei Maruoka, Mitsuru Esaki, yosuke Minoda, Noriko Tokunaga, Kazuhiro Haraguchi, Eikichi Ihara, Yoshihiro Ogawa

**Affiliations:** 1 91356 Department of Gastroenterology, Harasanshin Hospital, Fukuoka, Japan; 2 12923 Graduate School of Medical Sciences, Department of Medicine and Bioregulatory Science, Kyushu University, Fukuoka, Japan

**Fig. 1:**
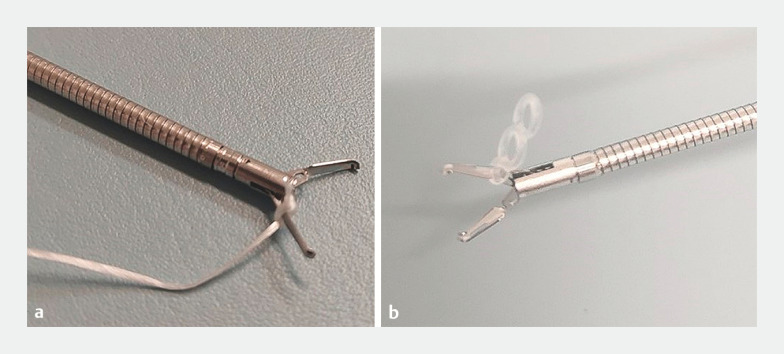
Clip with traction band and pre-tied thread.

Endoscopic submucosal dissection (ESD) is the standard treatment for early gastric neoplasms (EGNs), and traction methods have been developed to make ESD safe and simple 1 2 3 . Applying appropriate traction provides better visibility of the submucosal layer and enables effective dissection 4 5 . We developed a novel three-point traction (TPT) method using a combination of clip-with-thread and clip-with-silicon band for gastric ESD. Here, we present a successful case of TPT-ESD for a large EGN ( Fig. 1 , Video 1 ).

Three-point traction method for endoscopic submucosal dissection using clip-with-thread and clip-with-silicon bands for large early gastric neoplasms.Video 1

**Fig. 2:**
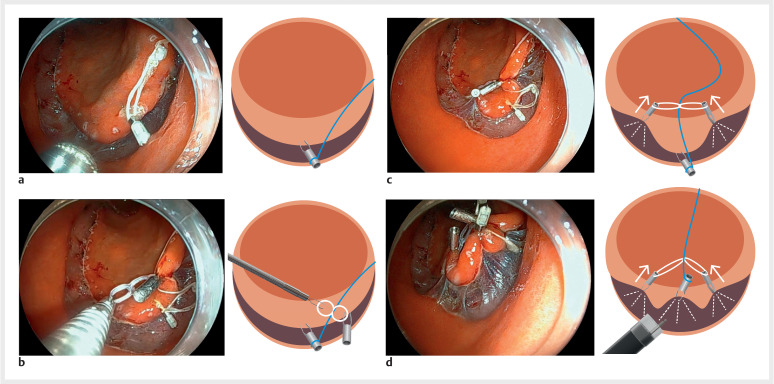
Each step of the three-point traction method for ESD using a combination of clip-with-thread and clip-with-silicon-band. **a** A clip-with-thread placed in the 6 o’clock position of the mucosal flap of the lesion. **b** A clip-with-silicon band placed in the 5 o’clock position of the mucosal flap. **c** The third clip placed in the 5 o’clock position of the mucosal flap while hooking the silicon band, allowing the thread to run underneath the silicon band (Fig. 2b and Fig. 2c). **d** The TPT force was achieved by pulling the thread using three clips.

A 70-year-old woman underwent ESD for a 50-mm EGN on the greater curvature of the antrum. Marking dots and circumferential mucosal incisions were made around the lesions using an electrosurgical knife. Subsequently, TPT was performed on the lesions. A clip-with-thread was placed at the 6 o’clock position of the mucosal flap of the lesion ( Fig. 2 **a** ). Next, a clip-with-silicon band was placed at the 5 o’clock position of the mucosal flap. The third clip was placed in the 7 o’clock position of the mucosal flap while hooking the band, allowing the thread to run underneath the silicon band ( Fig. 2 **b** , Fig. 2 **c** ). TPT force was achieved by pulling the thread using three clips ( Fig. 2 **d** ), which provided a wide and clear view of the submucosal layer, enabling stable submucosal dissection. En bloc resection was achieved without complication.

Compared with the conventional one-point-traction using a clip-with-thread, the TPT method provides a wide and clear view of the submucosal layer, elevating a larger area of the lesion and preventing the lesion from twisting during the latter part of dissection. The silicon band traction force generated by two clips in the 5 and 7 o’clock positions of the mucosal flap can bring the submucosal layer toward center, generating a synergistic effect with the TPT force. TPT- ESD can be a treatment option for EGN, particularly large lesions.
